# Weakly-Activated Phenol Derivatives as New Green Electrophilic Partners in Suzuki–Miyaura and Related C-C Couplings

**DOI:** 10.3390/molecules30010051

**Published:** 2024-12-26

**Authors:** Jules Perney, Alexandre Humblot-Negri, Carlos Vaca-Garcia, Sébastien Lemouzy, Martine Urrutigoïty

**Affiliations:** 1 Laboratoire de Chimie Agro-Industrielle, INRAE, Toulouse-INP, Université de Toulouse, 31030 Toulouse, France; jules.perney@etu.toulouse-inp.fr (J.P.); alexandre.humblotnegri@etu.inp-ensiacet.fr (A.H.-N.); carlos.vacagarcia@ensiacet.fr (C.V.-G.); 2 Laboratoire de Chimie de Coordination, CNRS, Toulouse-INP, Université de Toulouse, 31000 Toulouse, France

**Keywords:** Suzuki–Miyaura coupling, cross coupling, phenol derivatives, homogeneous catalysis, green chemistry

## Abstract

In recent years, there has been growing interest in the development of greener alternatives to traditional reagents used in carbon–carbon coupling reactions, particularly in response to environmental concerns. The commonly used aryl halides, despite being highly reactive in the Suzuki–Miyaura coupling (SMC), pose significant environmental risks. As a result, research has shifted towards exploring the use of phenols, which are widely accessible and environmentally benign. However, phenols are considerably less reactive due to the poor leaving group properties of the hydroxyl group, necessitating prior activation to facilitate their use in coupling reactions. This work aims to review the recent investigations on the activation strategies for phenols, focusing on their application in the Suzuki–Miyaura and related C-C couplings. In addition, the exploration of the potential of conducting the activation step “in situ” will also be discussed. We hope that this article will pave the way for the development of more sustainable and efficient coupling methodologies, addressing both ecological and practical challenges in organic synthesis.

## 1. Introduction

**Figure 1:**
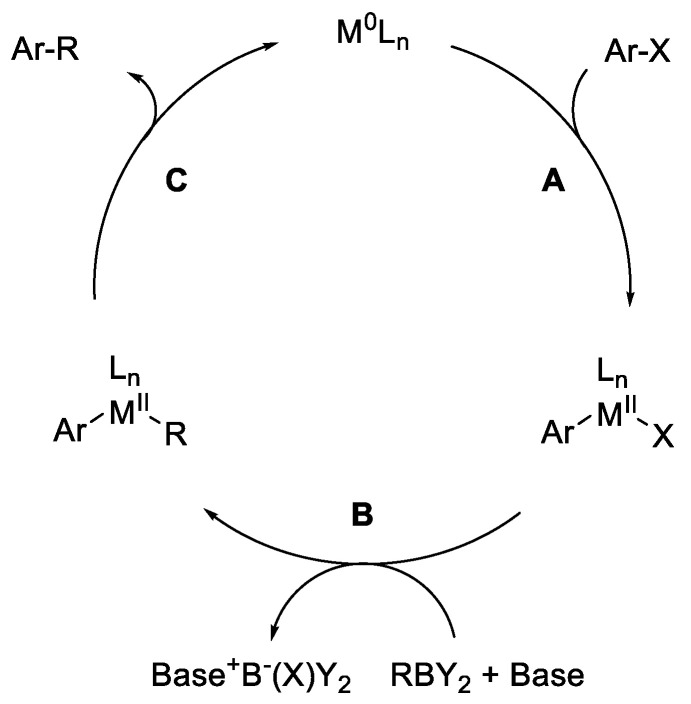
The catalytic cycle of the Suzuki reaction with aromatic electrophiles.

The Suzuki–Miyaura coupling (SMC) reaction is a widely employed method in organic chemistry for the formation of carbon–carbon bonds [1]. This reaction involves the cross-coupling of aryl or vinyl boronic acids with aryl or vinyl halides or triflates, catalyzed by palladium complexes. Introduced by Akira Suzuki and Norio Miyaura in 1979 [2], this reaction has since become a cornerstone in the synthesis of biaryl compounds, which are prevalent in pharmaceuticals [3], agrochemicals [4], and organic materials [5]. In a typical Suzuki coupling (Figure 1), the reaction mechanism is initiated by the oxidative addition of the aryl halide to the palladium (0) or nickel (0) catalyst (step A), forming a palladium (II) or nickel (II) complex. The next step involves the transmetalation process (step B), where the R’ carbon group from the boronic acid is transferred to the palladium center. Finally, reductive elimination occurs (step C), leading to the formation of the desired coupling product alongside the regeneration of the catalytically active low valent metal complex. The Suzuki–Miyaura coupling is mild, versatile, and makes use of stable, non-poisonous boronic acids, making it extremely appealing from a synthetic standpoint. It tolerates many useful functional groups, and ligand design has allowed for its performance and selectivity improvements [6,7,8,9].

**Figure 2:**
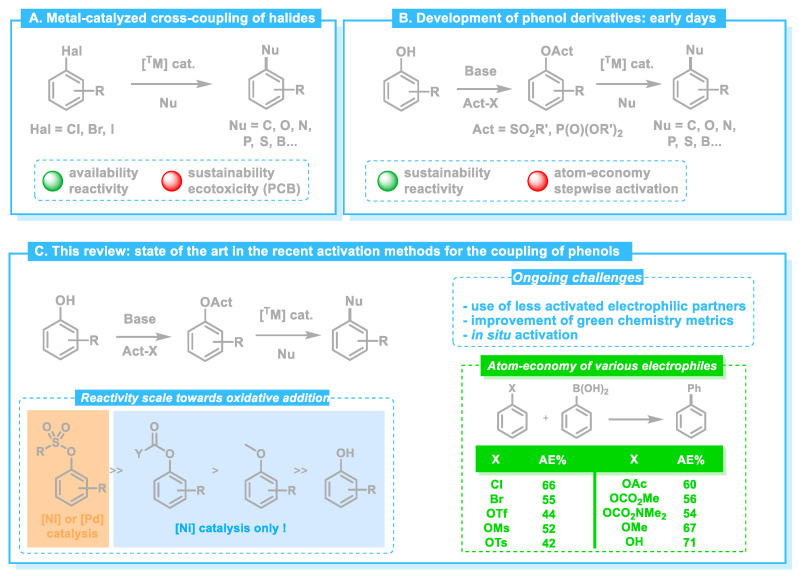
Development of phenol derivatives as electrophilic partner in cross-coupling reactions.

Usual catalytic systems involve palladium or nickel complexes bound to either phosphine or N-heterocyclic carbenes as the ligands. Furthermore, both aryl [10] and alkyl [11] boron reagents may be used as the nucleophilic partner in the process. The high yields, wide substrate versatility, and functional group tolerance of the Suzuki–Miyaura coupling have made it an indispensable tool in both academic and industrial settings [12]. Its application ranges from the synthesis of complex natural products to the production of key intermediates, for example, in drug development [3]. However, despite its widespread use, the reaction still poses some challenges, particularly in terms of the environmental impact. Indeed, the traditional reliance on aryl halides (Figure 2A) in Suzuki reactions poses environmental concerns due to the hazardous nature of these compounds along with the use of precious palladium complexes as catalysts.

These challenges have prompted ongoing research into developing greener alternatives and improving the sustainability of the Suzuki–Miyaura reaction [13]. To that end, phenol derivatives have gathered significant attention (Figure 2B) as potential substrates for Suzuki–Miyaura coupling, especially in the context of developing greener and more sustainable chemical processes. Indeed, phenolic compounds are ubiquitous in nature, particularly in plants, where they serve as essential building blocks. They play a crucial role in lignin, especially in the form of guaiacol derivatives. Phenolic structures are also found in certain vitamins, such as tocopherol, as well as in hormones (steroid-like estrone and its derivatives) and amino-acids (tyrosine being a notable example). Consequently, researchers have been exploring the use of phenol-based derivatives, which are, unlike aryl halides, widespread in nature and environmentally benign. However, the main challenge with phenols lies in their poor reactivity (especially towards oxidative addition, see Figure 1, step A), requiring prior activation of the hydroxyl group to form more reactive intermediates. Several phenol derivatives have been studied for their potential application in Suzuki–Miyaura coupling. The use of phenol phosphates and sulfonates (Figure 2B) as the electrophilic partner is beyond the scope of this review, as these substrates have been extensively reviewed in the last decade [14,15]. This review will focus on greener alternatives, featuring less activated C-O bonds on the phenolic ring (Figure 2C), ranging from carboxylates to the direct coupling of phenols via in situ activation. Compared to aryl sulfonates, for which the oxidative addition may be effected by a palladium (0) complex as the catalyst, it seems that more reductive nickel (0) is necessary for this step to proceed in the case of less reactive aryl esters, carbonates, carbamates, and ethers (Figure 2C) [16,17,18]. However, despite the challenges that less activated phenolic compounds present, they have great potential to improve the green aspect of these cross-coupling reactions.

Figure 2C (bottom right) summarizes the atom economy of the Suzuki–Miyaura reaction with a range of phenol derivatives as electrophilic partners to provide biphenyl, using phenylboronic acid as the pro-nucleophile. While highly reactive sulfonates (OTf, OMs, and OTs) result in low atom economy (42–52%, compared to the benchmark aryl bromides with 55% atom economy) given that they produce stoichiometric amounts of high-molecular-weight sulfonates as the byproducts; phenols substituted by smaller, less-activated leaving groups (especially OAc, OCO_2_Me, and OMe) feature much higher atom economy (56–67%). Carbonates and carbamates activating groups have even more potential in that regard if one can manage to recycle the CO_2_ produced throughout the coupling process [19].

Therefore, this review will cover the recent advances in the Suzuki–Miyaura coupling using weakly activated phenolic derivatives, including the following:

▪ O-aryl esters;
▪ Aryl carbonates;
▪ Aryl carbamates;
▪ Aryl ethers.

In addition, the in situ activation enabling the direct use of phenols as substrates in such coupling reactions will be discussed. Although the Suzuki–Miyaura coupling is the main topic of this review, the related Miyaura borylation as well as other C-C coupling reactions (such as Negishi/Kumada couplings and miscellaneous C-C coupling reactions) will also be discussed.

## 2. O-Aryl Esters (Aryl Carboxylates)

**Figure 3:**
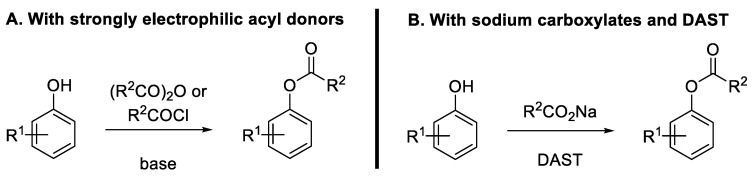
Methods for the synthesis of O-aryl esters from phenols.

Aryl carboxylates (also known as O-aryl esters) are usually prepared by the esterification of phenols with strong electrophilic acyl donors, such as acyl chlorides or carboxylic acid anhydrides (Figure 3A). However, a greener alternative has recently emerged to produce such compounds by reacting phenols and sodium carboxylates at room temperature using Diethyl Ammonium Sulfur Trifluoride (DAST) as the activating agent (Figure 3B). The reaction proceeds via the in situ generation of an acyl fluoride species [20].

**Figure 4:**
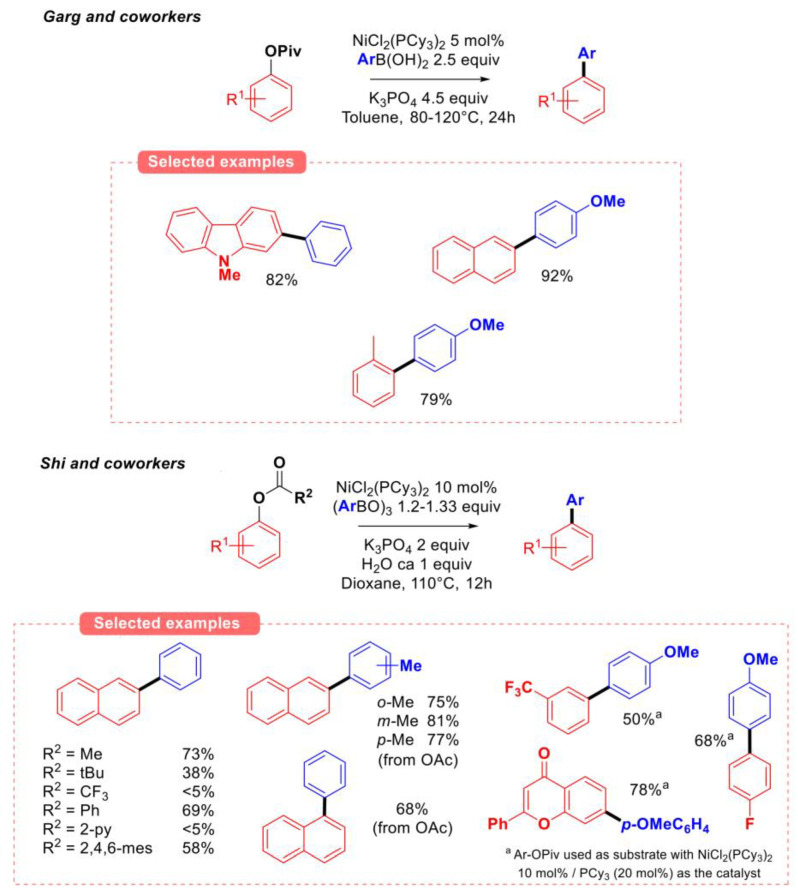
Initial reports on the Suzuki–Miyaura coupling of O-aryl esters, Garg group [21], Shi group [22].

The reactivity of O-aryl esters in Suzuki–Miyaura coupling conditions has been simultaneously reported in 2008 by the Garg group using boronic acids [21] and by the Shi group using tri-arylboroxines [22] as the nucleophilic partners (Figure 4).

In both cases, NiCl_2_(PCy_3_)_2_ was found to be a superior catalyst when compared to nickel (II) complexes featuring other phosphine or phosphite ligands. However, PdCl_2_(PCy_3_)_2_ was found to be ineffective under these conditions. The scope of O-aryl esters has been studied, and it was shown that the nature of carbon group (R_2_) on the carboxylate greatly influences the reactivity of the electrophile [22]. Indeed, both aryl and small alkyl groups resulted in high reactivity of the ester precursor towards oxidative addition of the Ni catalysts in the aryl C-O bond. More activated precursors (R_2_ = CF_3_ or 2-pyridyl) resulted in no conversion due to the complete hydrolysis of the starting material under basic aqueous conditions. In the case of OPiv as the leaving group (R_2_ = *t-*Bu), the hydrolysis was shut down, but the rate of the coupling process dropped significantly.

Mechanistic investigations of the coupling reaction of phenyl acetate and phenyl boronic acid by DFT performed by Liu and coworkers revealed that the transmetalation step is likely to be rate-limiting. Furthermore, the authors identified that in the case of the OAc leaving group, the oxidative addition of the acyl C-O bond is kinetically favored compared to that of the aryl C-O bond. However, the oxidative addition in the acyl C-O bond is, unlike that of the aryl C-O bond, reversible and presents a higher barrier towards transmetalation [23]. This hypothesis could explain the observed drastic effect of the carboxylate substituents (Figure 4) [22], for which the potential acceleration/irreversibility of the oxidative addition of Ni (0) in the acyl C-O bond due to electronic effects (R_2_ = CF_3_) or pre-coordination (R_2_ = 2-pyridyl) might account for the observed fast hydrolysis.

**Figure 5:**
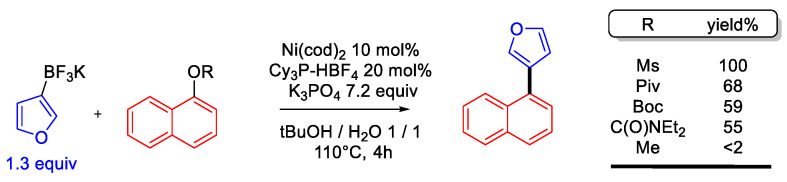
SMC of naphthyl pivalates with potassium (aryl)trifluoroborates [19].

The Suzuki coupling of naphthyl pivalates with potassium heteroaryl trifluoroborates has also been investigated using a similar catalytic system (in situ-generated Ni^0^(PCy_3_)_2_ from Ni(cod)_2_) in tBuOH/water mixture as the solvent) [24]. The initial screening of the naphthyl oxygenated leaving group suggests that while naphthyl pivalates are slightly more reactive than their carbonate and carbamate analogs, they are much less reactive than naphthyl mesylates. This reactivity trend was confirmed by comparing the reactivity of naphthyl mesylate (64–93% yield) and pivalate (26–78% yield) with a series of heteroaryl trifluoroborates. It is worth noting that naphthyl methyl ether was not reactive under these conditions (Figure 5).

**Figure 6:**
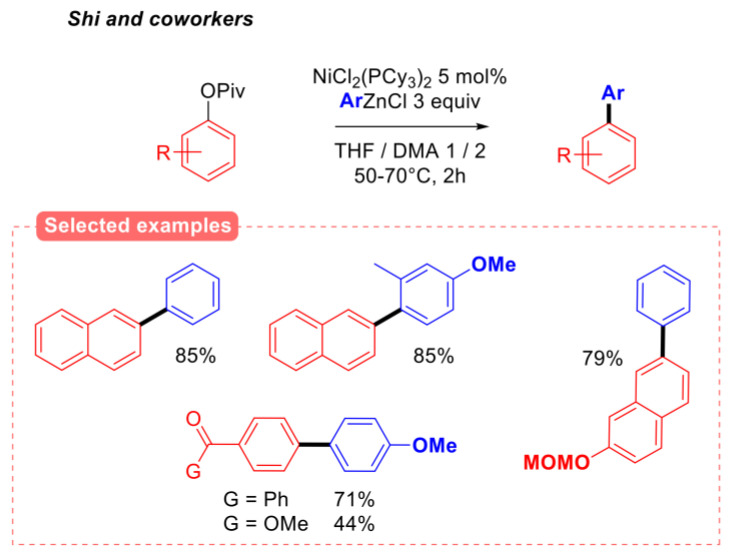
Aryl–aryl Negishi coupling of aryl pivalates, Shi group [25].

Furthermore, unlike previous reports [21,22], nickel (II) complexes were not found to be effective pre-catalysts for this reaction, presumably due to the slower generation of the catalytically active Ni(0) species from Ni(II) by the potassium heteroaryl trifluoroborate, when compared to aryl boronic acids and esters. The aryl–aryl Negishi cross-coupling of aryl pivalates was also reported by the Shi group (Figure 6) [25]. As for boron nucleophiles, the reaction of aryl zinc chlorides tolerated both naphthyl and phenyl pivalates as the electrophilic partner. Interestingly, this reaction was also found to be compatible with electrophilic carbonyl substituents, such as ketones.

**Figure 7:**
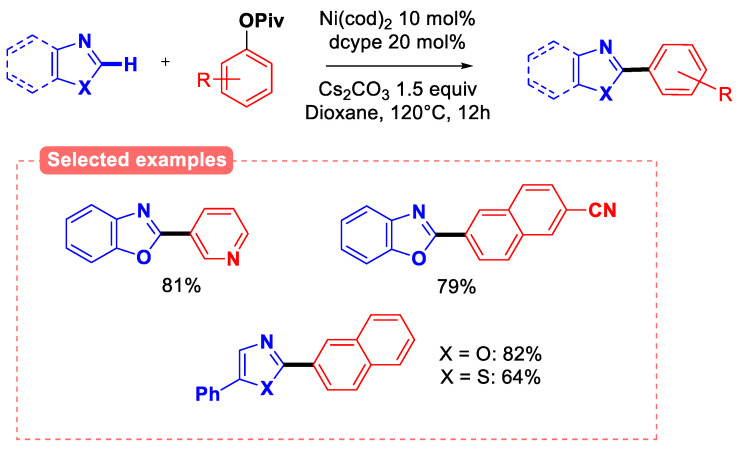
Direct C-H arylation of azoles with aryl pivalates.

Finally, aryl carboxylates were further used to develop direct C-H arylation methods using azoles as aryl C-H pronucleophiles (Figure 7) [26].

Indeed, both aryl and naphthyl pivalates were successfully coupled with a diversity of azoles C-H bonds. Mechanistic investigations on isolated naphthyl Ni (II) (dcype) pivalate complex (dcype = 1,2-bis(dicyclohexylphosphino)ethane) revealed the importance of the pivalate anionic ligand. Indeed, the kinetic study of the reaction shows zero order vs. the aryl pivalate, while it features first order in both the azole and the ArylNi(II) pivalate catalytic intermediate, suggesting that the concerted metalation–deprotonation (CMD) process is the rate-determining step. This hypothesis was supported by the high kinetic isotopic effect (KIE = k_H_/k_D_ = 2.4) observed for the aryl C-H/D bond of the azoles partner [27].

## 3. Aryl Carbamates

**Figure 8:**
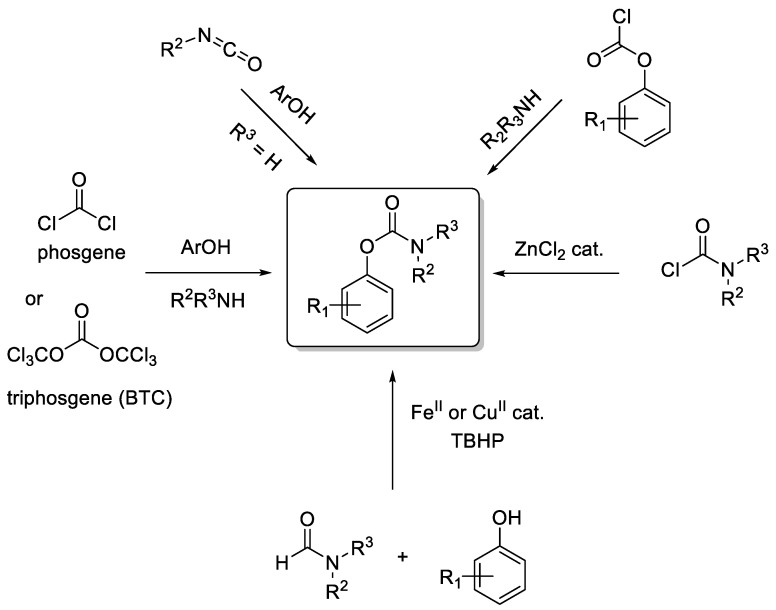
Preparation of O-aryl carbamates.

Aryl carbamates represent an important structural motif for the synthesis of drugs and other industrial chemicals. These compounds can be synthetized from phenols using a variety of methods (Figure 8) [28,29]. A particularly effective route is based on the reaction of phosgene or substitute (such as triphosgene, also known as BTC: bis-trichloromethyl carbonate) in the presence of a phenol and an amine [28,29]. In this reaction, the alcohol reacts with phosgene to form the corresponding aryl chloroformate in situ, which then reacts with the amine to generate the desired carbamate. A similar stepwise process starting from isolated chloroformates in the presence of a primary or secondary amine in basic conditions [30] is also possible. Similarly, the carbamate product may be prepared by reacting N,N-dialkyl carbamoyl chlorides under Lewis acid [31] or Lewis basic [32] conditions. Carbamates can also be produced by reacting alcohols in the presence of an isocyanate as the electrophile [33,34]. However, all these methods suffer from their reliance on the very toxic phosgene or BTC, as these reagents (isocyanates, O-aryl chloroformates, and N,N-dialkyl carbamoyl chlorides) are prepared from phosgene or triphosgene. Therefore, new synthetic routes are gradually being explored with the aim of limiting the use of toxic compounds. Among these, the copper [35]- or iron [36]-catalyzed oxidative coupling reaction of phenols with less toxic formamides (used as the solvent) is a notable improvement to achieve the synthesis of O-aryl carbamates.

**Figure 9:**
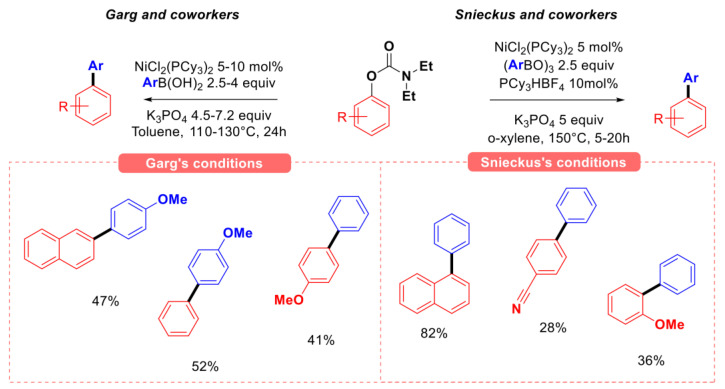
Seminal applications of aryl carbamates as substrates for SMC, Garg [37] and Snieckus [38].

In 2009, Garg [37] and Snieckus [38] independently explored the reactivity of O-aryl N, N-diethyl carbamates in Suzuki–Miyaura coupling (SMC) conditions using a catalytic system based on NiCl_2_(PCy_3_)_2_ and potassium phosphate as the base (Figure 9). Garg’s group demonstrated the ability to couple phenol derivatives carbamates with aryl boronic acids, obtaining moderate yields up to 52% after 24 h. Notably higher yields were obtained when coupling naphthol derivatives. Snieckus’s group-modified version of the reaction (by replacing boronic acids with tri(aryl)boroxin as the nucleophilic partner and using PCy_3_HBF_4_ as additive) afforded slightly better yields, especially for phenyl carbamate substrates. The coupling of aryl N,N-dimethyl carbamate electrophiles with tri(aryl)boroxin was also studied later by Shi using similar conditions in dioxane as the solvent at 110 °C, obtaining *para-*substituted biphenyl derivatives in increased yield (62–85%) [39].

**Figure 10:**
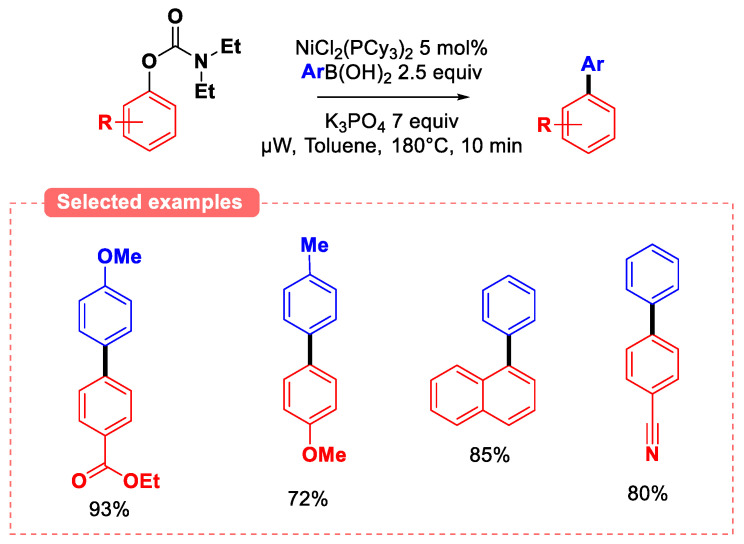
Microwave activation in the Suzuki–Miyaura coupling of carbamates.

Later, Garg and Snieckus reported a combined experimental and computational study on the cross-coupling of O-aryl carbamates with aryl boronic acids [40]. Indeed, using 2.5 equivalents of a 10:1 mixture of triarylboroxin/arylboronic acid as the nucleophilic partner in conditions similar to Snieckus’ initial article [38] resulted in slightly improved yields of the biaryl products when compared to the initial reports. Computational study suggests that the transmetalation step is likely to be rate-determining. Building on these works, Kappe and his team explored the effect of microwave activation [41] in conditions similar to Garg’s initial work (Figure 10). The use of microwaves enabled a significant increase in reactivity, and yields of up to 93% were achieved within 10 min. All these studies showed that electron-withdrawing substituents increase the reactivity of phenolic carbamates, probably owing to the enhancement of the supposed rate-determining oxidative addition step.

**Figure 11:**
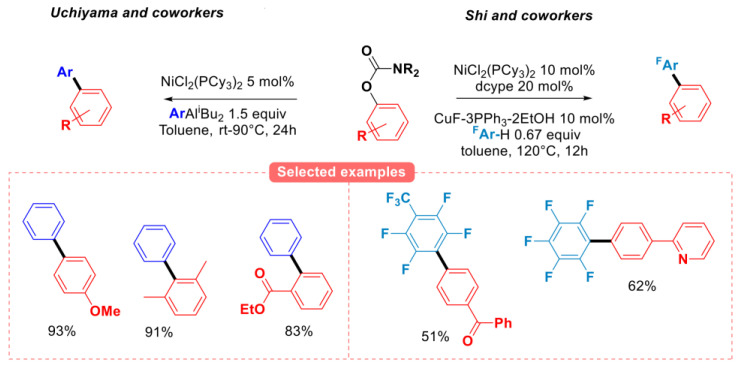
Miscellaneous C-C coupling with N,N-dialkyl O-aryl carbamates, Uchiyama [42], Shi group [43].

Other groups have also investigated the reactivity of aryl carbamates for developing miscellaneous C-C coupling reactions (Figure 11). Uchiyama and coworkers explored the reactivity of N,N-diethyl O-aryl carbamates with (di-*i-*butyl) aryl aluminum species using NiCl_2_(PCy_3_)_2_ as the catalyst, affording good to excellent yields of a variety of diversely functionalized biaryl compounds [42]. The direct C-H arylation of N, N-dimethyl O-aryl carbamates with perfluoroarenes was realized by the Shi group, using CuF-3PPh_3_-2EtOH as the co-catalyst, probably through the formation of a transient perfluoroaryl copper(I) species [43].

## 4. Aryl Carbonates

**Figure 12:**
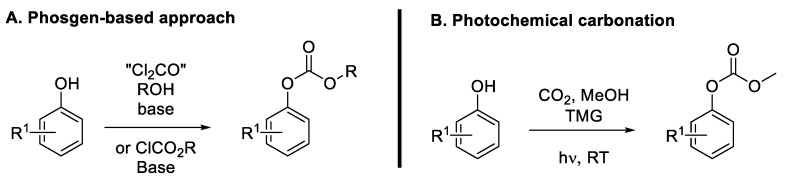
Preparation of aryl carbonates.

Aryl carbonates could be considered among the most promising phenol derivatives for coupling reactions, offering a good balance between reactivity and environmental friendliness [44]. Like esters and carbamates, the carbonate group is a relatively good leaving group, owing to the weakening of the aryl C-O bond due to the electron-withdrawing ability of the alkyloxycarbonyl moiety. This activation facilitates the oxidative addition step. Like carbamates, aryl carbonates have long been synthetized using phosgene or chloroformates (Figure 12A) [44]. These reagents can react with alcohols to obtain the corresponding carbonate with high yields. However, the high toxicity of these phosgene-derived reagents used for these syntheses is a major hindrance, especially for industrial applications. In addition, aryl alcohols react much slower with phosgene than alkyl alcohols, and therefore, harsher conditions (high temperatures, excess of pyridine base) are usually required to ensure the efficiency of the process. Recently, new efficient methods to synthetize aryl carbonates from phenols using a greener approach have emerged. For instance, the use of CO_2_ for photocatalyzed transformation of phenols (Figure 12B) [45] and trans-carbonation between aryl alcohols and dialkyl carbonates [46] are the most notable examples.

**Figure 13:**
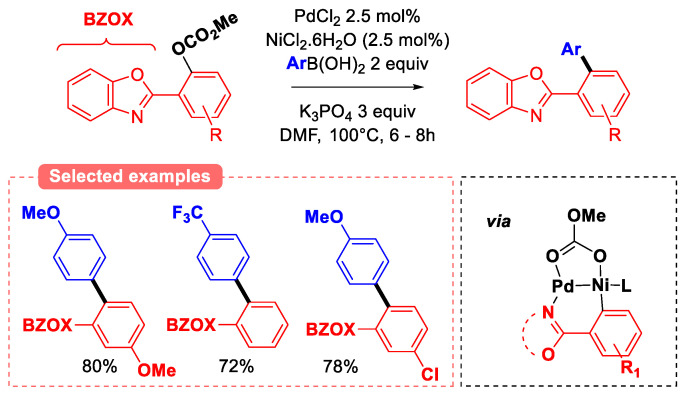
Reactivity of benzoxazole-substituted aryl carbonates.

Aryl *tert-*butyl carbonate’s reactivity for Suzuki cross-coupling was first studied using NiCl_2_(PCy_3_)_2_ catalytic system and K_3_PO_4_ as a base [37]. Naphthol derivatives were converted with good to high yields, but unfortunately, no experiments have been conducted with phenol derivatives. Similarly, the cross-coupling of sterically hindered aryl carbonates with aryl boronic acids was also explored (Figure 13) [47]. The authors investigated a different catalytic approach, focusing on the use of bimetallic Ni-Pd nanoparticles and aryl carbonate substrates featuring a nitrogen chelating group (benzoxazole) *ortho* to the carbonate leaving group. High yields were obtained in relatively short reaction times (6–8 h), which can be explained by the dual Ni/Pd catalytic activation of the substrate via coordination of the tethered benzoxazole moiety (see Figure 13 bottom right). Indeed, it was proposed that Ni coordination to the hindered oxazolidinone enhances the rate of the rate-limiting oxidation addition step.

**Figure 14:**
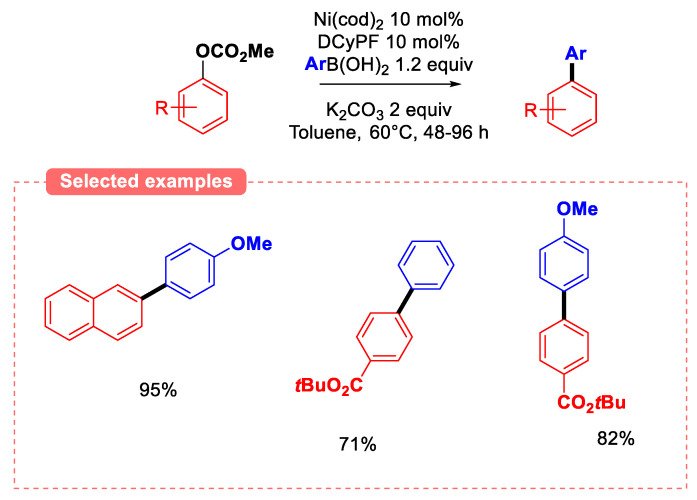
Ni-catalyzed SMC of aryl methyl carbonates.

Based on Garg’s conditions, a new catalytic system that allows for the coupling of coordinating group-free methyl carbonates derived from phenols was developed (Figure 14) [48]. Catalyst screening identified Ni(cod)_2_-DCyPF (DCyPF = 1,1′-bis(dicyclohexylphosphino)ferrocene) as the only effective complex for the coupling of *para-*methoxyphenyl carbonate with phenyl boronic acid. The study on other carbonate substrates revealed that the highest yields were obtained when electron-withdrawing groups were introduced at the *para*-position of the phenyl ring. Likewise, the presence of electron-donating substituents on the aryl ring of the boronic acid afforded high yields. However, this approach suffers from very long reaction times (typically in the 48–96 h range).

## 5. Aryl Ethers

### 5.1. Anisoles and Other O-Alkyl Ethers

**Figure 15:**
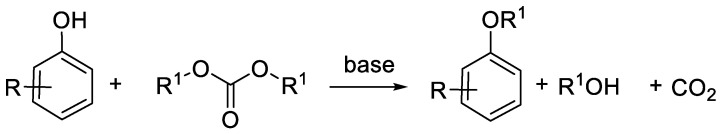
Methylation of phenols by dialkyl carbonates.

Generally, aryl methyl ethers (also known as anisoles) are synthesized by the O-methylation reaction from phenols with methyl halides [49,50,51] or dimethylsulfate [52,53]. However, these methods use harmful reagents and require a stoichiometric amount of strong base to form the phenolate and neutralize the acid by-product. Large quantities of inorganic salts must be eliminated. A more environmentally friendly reaction was the use of carbonates, such as DMC (dimethylcarbonate), as a methylating agent. DMC and other dialkyl carbonates offer powerful perspectives for the development of alkylation processes having a low environmental impact. Furthermore, only methanol and CO_2_ by-products are produced throughout this process [54,55] (Figure 15).

The O-methylation of phenols is generally carried out in a reactor at high temperatures between 120 and 200 °C in the presence of alkali or organic bases used as catalysts. Basic zeolites, alumina, or alumina-silica have also proven to be good catalysis in a continuous process. High yields of anisoles were achieved but in the presence of by-products C-methylation [56,57]. The continuous-flow process under gas–liquid phase transfer catalysis conditions (GL-PTC) involving PEG (polyethylene glycol) and the carbonate potassium base has been described [58,59,60,61,62]. In addition, an effective semi-continuous process was reported using tetrabutylammonium bromide (TBAB) as a base [63]. The reaction was carried out in the absence of a solvent at a temperature higher than the boiling point of DMC under atmospheric pression. Various phenol derivatives were treated and very good yields (67–100%) with excellent O-methylation selectivity (98–100%) were obtained. As the thermal stability of TBAB could represent a problem, the authors carried out the same study using more thermally stable K_2_CO_3_ as an inorganic base instead of TBAB [55]. By operating at an optimum temperature of 160 °C and an optimum substrate/K_2_CO_3_ ratio in order to maintain homogeneous conditions, the phenols studied were transformed with excellent conversions and selectivities. Diethylcarbonate (DEC) was also involved in the reaction giving a 90% yield in O-ethylation of phenol and *p-*cresol.

A N,N-dimethylformamide/dimethylacetal (DMF/DMA) mixture was also employed as a methylating agent to successfully transform various para-substituted phenols under microwave irradiation. The yields of para-substituted anisoles obtained ranged from 69 to 98%. The reactions had to be carried out at a maximum power of 500 W in order to avoid any risk of explosion at higher powers in the microwave, with reaction times of 30 min with electron-withdrawing substituents or 60 min with electron-donor groups [64].

**Figure 16:**
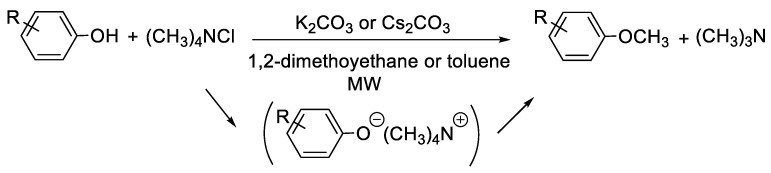
Methylation of phenol derivatives using (CH_3_)_4_NCl as methylating agent.

Another alternative to the use of methylating agents has been reported with ammonium salts [65]. The microwave-assisted O-methylation of various phenolic compounds using a slightly excess of tetramethylammonium chloride and potassium carbonate as a base in heterogeneous conditions afforded moderate to excellent yields (45–96%) that can generally be improved by using cesium carbonate when required. The secondary products of the reaction were trimethylamine, which is a gas and easily eliminated, and the chloride salt of the base used (Figure 16).

**Figure 17:**
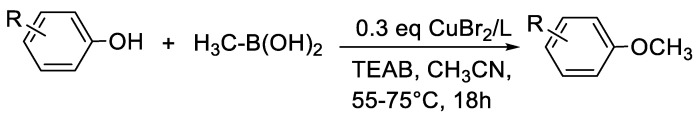
The Cu-catalyzed phenol O-methylation reaction.

More recently, O-methylation was developed by the Cu-catalyzed oxidative cross-coupling of various phenols in the presence of methylboronic acid allowing for the corresponding aryl methyl ethers. The chosen catalytic system was CuBr_2_/2,2-bipyridine or 1,10-phenathroline ligand/tetraethylammonium bicarbonate (TEAB) base [66] (Figure 17).

Phenolic derivatives with electron-withdrawing substituents are transformed in high yields up to 99%. However, electron-rich phenol derivatives proved to be poor substrates for methylation, and it is the phenol homodimerization reaction that takes place to give the by-products.

**Figure 18:**
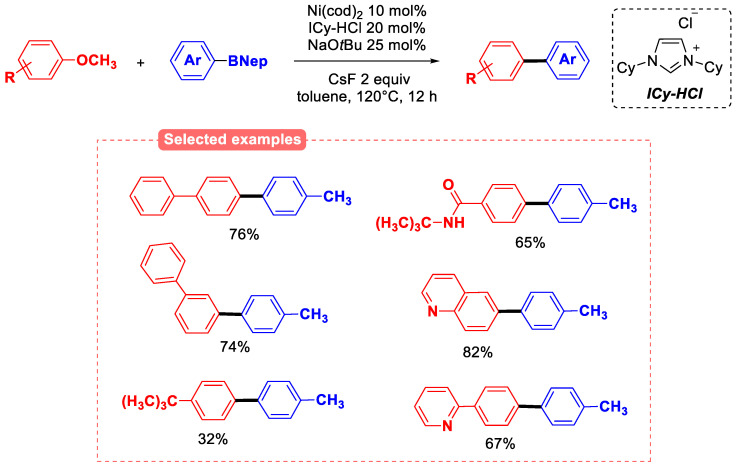
The Suzuki–Miyaura reaction of aryl methyl ethers catalyzed by Ni and ICy ligand.

The development of a nickel-based catalyst for the Suzuki–Miyaura-type reaction of a range of aryl methyl ethers has led to successful results, even with heteroaryl derivatives. These include the use of various heterocyclic carbenes that should facilitate an oxidative addition due to their very strong electron-donating properties. Although naphthyl methyl ethers can be efficiently coupled with boronic esters in the presence of Ni(PCy_3_)_2_ as the catalyst [67], phenyl ethers are less reactive towards the rate-limiting oxidative addition step due to the higher aromatic stabilization of the benzene ring when compared to the naphthyl one. Therefore, catalyst screening revealed that very strongly donating cyclohexyl-substituted carbene ligand ICy was optimal in the case of monocyclic aryl methyl ethers, while Ni(PCy_3_)_2_ failed to yield the coupling product. Among the tested N-heterocyclic carbene (NHC) ligands, only unsaturated NHCs bearing a secondary alkyl group as the nitrogen substituent were effective. The Ni(cod)_2_/ICy system catalyzed the cross-coupling of aryl methyl ethers with organoboron compounds in good to excellent yields [68]. The study with several different aryl ethers and boronic esters allowed for the cross-coupling product to be obtained. Electron-rich and electron-deficient boronic esters favorably gave the cross-coupling reaction with methoxynaphthalene in CsF-free conditions. In addition, an anisole derivative and a heteroaromatic substrate can be coupled to form the corresponding biaryl products. (Figure 18). The use of the ICy ligand thus ensured the cross-coupling of a range of methoxyarenes and arylboronic esters in the absence of CsF [69]. Mechanistic studies on the reaction has been reported, showing that in the nickel-catalyzed reactions, the oxidative addition of the C(aryl)−OMe bond can proceed more easily without the aid of CsF because the nickel-ligand bonds are stronger and therefore stabilize the transition state. The subsequent transmetalation from an Ar−Ni−OMe intermediate is determined to proceed through a pathway with lower energies than those required for β-hydrogen elimination. The overall driving force of the reaction is the reductive elimination to form the carbon−carbon bond [69]. Similarly, the homocoupling of various phenols and naphthols was enabled via a Miyaura borylation/Suzuki–Miyaura coupling sequence under the same conditions [70].

**Figure 19:**
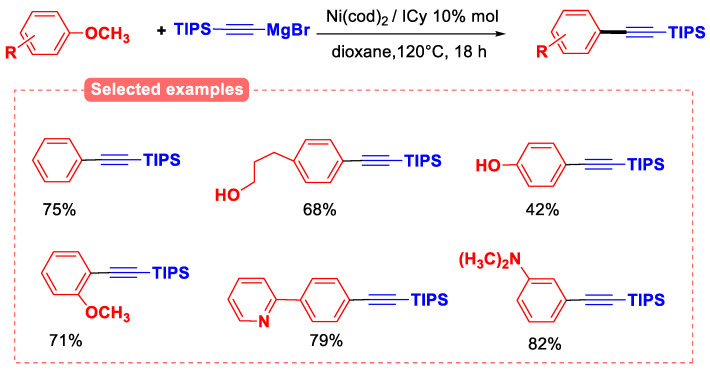
The cross-coupling reaction between anisole derivatives and the alkynylmagnesium compound.

Another interesting study was reported using the same catalytic system for the cross-coupling reaction between anisoles and C(sp)−nucleophiles for the construction of C(sp^2^)−C(sp) bonds (Figure 19) [71]. The work was focused on using the alkynylmagnesium compound (alkynyl-MgX) generated by the reaction of ethynyltriisopropylsilane with ethylmagnesium bromide. The reaction was conducted with 2 eq. of alkynyl-MgBr in dioxane for 18 h. The steric hindrance of the triisopropylsilyl (TIPS) group proved to be crucial for the success of the reaction. It is important to note that the TIPS group can then be easily eliminated to give the corresponding terminal alkynes, which are interesting as building blocks for the synthesis of more complex molecules.

The scope of aryl ether substrates was studied, and the reaction was compatible with free alcohol and phenol substituents, although 3 equiv of the Grignard reagent was used in these cases. A similar Kumada-type coupling reaction has also been reported using hindered alkyl Grignard reagents with anisoles using the Ni^0^/ICy catalytic system at 80–140 °C [72].

**Figure 20:**
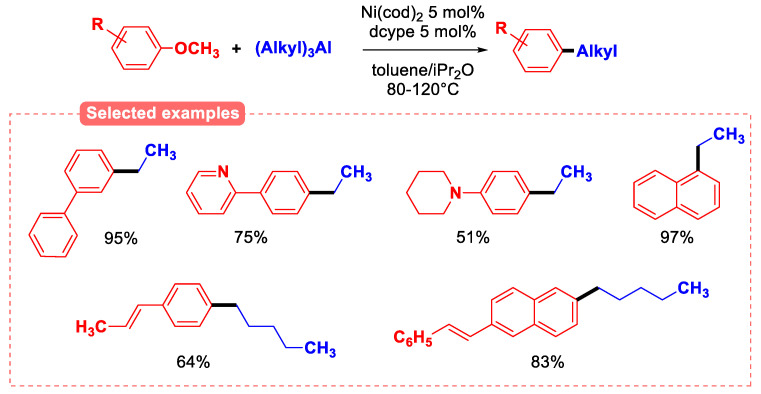
The aryl ether alkylation reaction with the activation of C-O bond by Lewis acidic trialkyl aluminum reagents.

Lewis acidic nucleophilic partners have been used to lower the activation energy of C(sp^2^)-OMe bond breaking by prior coordination. The activation of the C-O bond by the strong and oxophilic Lewis acid trialkylaluminium was demonstrated in the alkylation reaction catalyzed by Ni(cod)_2_ in the presence of the dcype ligand (Figure 20). A wide variety of aryl methyl ethers were studied in this C(sp^2^)-Csp^3^ coupling reaction, and the results gave very good yields of the corresponding products. Interestingly, a natural phenol derivative such as anethole was also successfully transformed in 64% yield [73].

Computational studies were conducted, and the calculations suggested that the oxidative addition is facilitated by the trialkylalumines by activating the C-O bond and the stability of the dialkylaluminium methoxide formed favors an efficient transmetallation step. The nickel catalyst can efficiently carry out the cycle to transform the substrates, thus suppressing β-H elimination, which is a competitor reaction, so high yields are achieved. Methylation of anisoles using Ni^0^/ICy as the catalyst using Me_3_Al at 80 °C has also been investigated [74].

### 5.2. 2-Pyridyl Ethers and Related Compounds

Compared to anisoles, phenols substituted by a 2-pyridyl offer higher reactivity towards an oxidative addition due to the electron-withdrawing ability of the pyridyl ring. Furthermore, the oxidative addition is enhanced by a coordination-directed process owing to the Lewis basicity of the *ortho* nitrogen atom. 2-Pyridyl ethers are usually prepared by nucleophilic aromatic substitution using 2-halo (usually chloro or fluoro) pyridines with phenolates generated in situ from the corresponding phenols in basic conditions.

**Figure 21:**
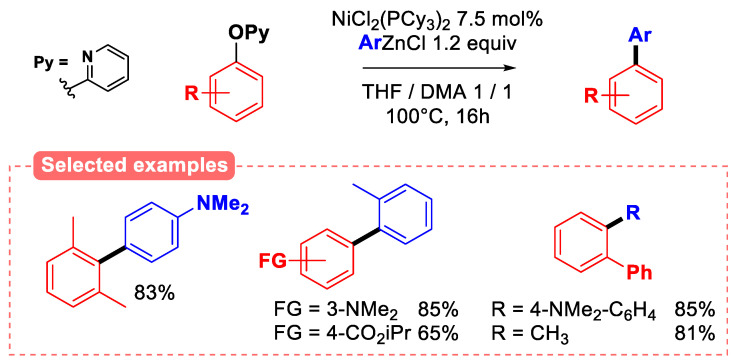
Negishi coupling of 2-pyridyl aryl ethers by Wang [75].

2-Pyridyl ethers have been investigated in a variety of coupling reactions, notably by the group of Zhong-Xia Wang. Indeed, the authors reported both the aryl–aryl and aryl–alkyl coupling of aryl 2-pyridyl ethers with a variety of organozinc reagents (Figure 21), using NiCl_2_(PCy_3_)_2_ as the catalytic system [75].

Interestingly, the reaction tolerates a wide array of electron-donating and electron-withdrawing groups on the phenolic moiety. In addition, sterically hindered *ortho-, ortho′*-disubstituted electrophiles could be used without damaging the efficacy of the process, and the reaction was amenable to the gram scale. Mechanistic investigations revealed that the 2-pyridyl group was essential to ensure the reactivity of the electrophile, as 3-pyridyl was unreactive in these conditions, and 4-pyridyl ethers resulted in poor conversion (18%), highlighting the importance of nitrogen coordination in the oxidative addition and/or transmetalation step. The Kumada coupling of 2-pyridyl ethers with aryl magnesium bromides has also been explored using CrCl_2_ as the catalyst, although mostly β-naphthol derivatives were used in this study [76].

**Figure 22:**
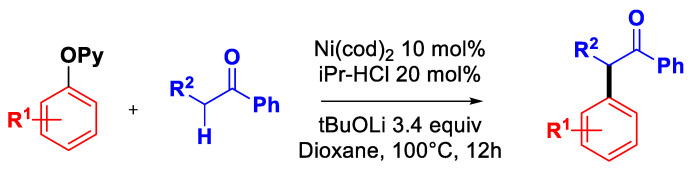
α-Arylation of ketones with 2-pyridyl aryl ethers by Wang’s group [77].

In addition, Wang’s group also reported the α-arylation of ketones with 2-pyridyl ethers using a Ni^0^-NHC complex as the catalyst (Figure 22) [77].

**Figure 23:**
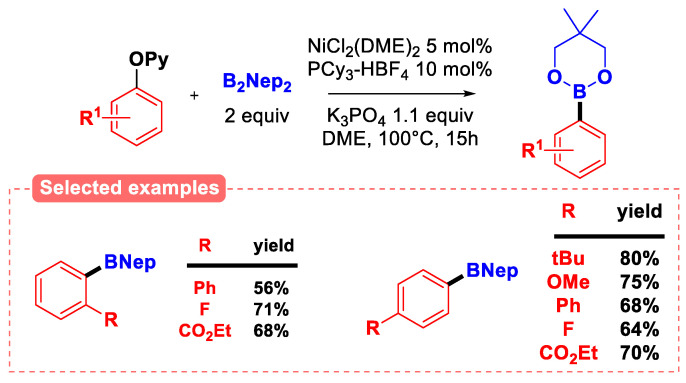
Ni-catalyzed borylation of 2-pyridyl aryl ethers [78].

Finally, Miyaura-type borylation has been achieved using either nickel (0) [78] or iron (II) [79] as the catalytic system. In particular, the nickel-catalyzed borylation of 2-pyridyl aryl (and benzyl) ethers developed by Chatani [78] proceeds using in situ generated Ni(PCy_3_)_2_ as the catalyst (Figure 23).

The reaction tolerates a large array of electron-donating and electron-withdrawing functional groups both in the *ortho-* and *para*-position. In the case of iron-catalyzed Miyaura borylation of 2-pyridyl aryl ethers [79], it was demonstrated (using a combination of EPR analysis, radical inhibition experiments, and radical clock experiments) that the reaction goes through a radical oxidative addition process, unlike the Ni-catalyzed version of the reaction, which is most likely operating via a two-electron process.

**Figure 24:**
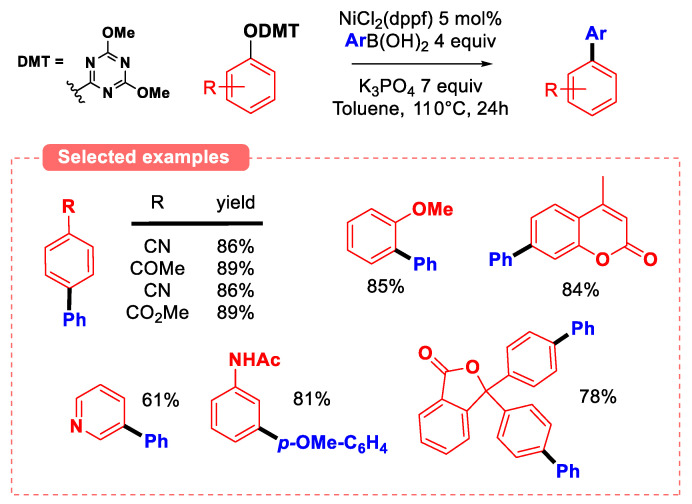
Suzuki–Miyaura coupling of ArODMT by Jin and coworkers [80].

The cross-coupling of aryl triazine ethers (2,4,6-triphenoxy-1,3,5-triazine) has also been thoroughly investigated. Seminal work by Jin and coworkers using 2-aryloxy-4,6-dimethoxy-1,3,5-triazines (2-ArODMT, prepared from the corresponding phenols and 2-chloro-4,6-dimethoxy-1,3,5-triazine under basic conditions) [80] as substrates in Suzuki–Miyaura coupling revealed that that such phenolic compounds are more prone to undergo oxidative addition than 2-pyridyl ethers (Figure 24). Indeed, less σ-donor dppf ligand was used when compared to methods featuring 2-pyridyl ethers (where PCy_3_ or NHC ligands are necessary for the reaction to proceed). In addition, the coupling of phenyl 2-pyridyl ether with *para-*methoxyphenyl boronic acid afforded the desired product in modest yield (26%), whereas 2-PhODMT afforded the same product in very high yield (90%) in the same conditions.

**Figure 25:**
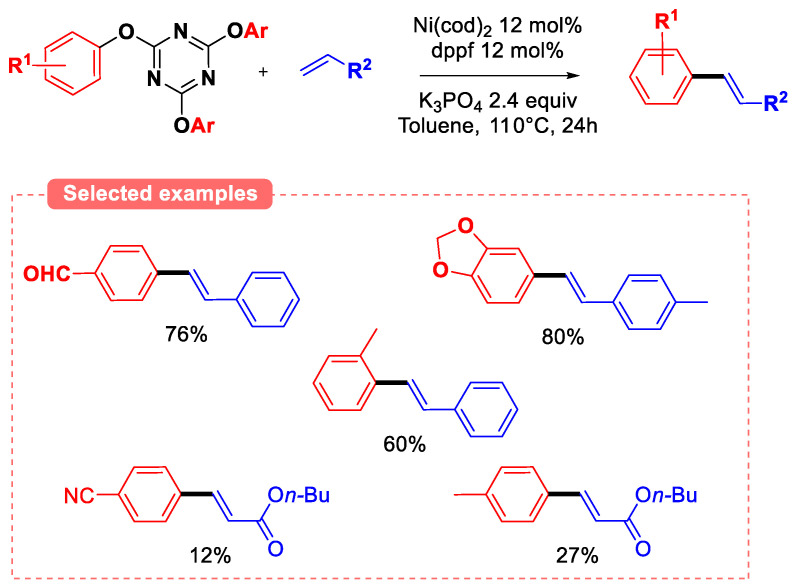
Heck coupling of tri(aryloxy) triazines.

The functional-group tolerance of this method is quite exceptional, as a diversity of functions (i.e., aldehydes, ketones, esters, nitriles, primary amides, halogens…) are inert to the reaction conditions. Furthermore, the tolerance of methoxy substituent on the phenolic ring suggests that this group does not undergo oxidative addition with Ni^0^(dppf), highlighting once more the increased reactivity of 2-pyridyl ethers derivatives when compared to anisoles. Given the success of ArODMT substrates in the Suzuki–Miyaura coupling reaction, 2,4,6-trichloro-1,3,5-triazine (TCT) has also been envisioned as an activating agent for the phenolic ring. The main advantage of preparing 2,4,6-triaryloxy-1,3,5-triazine from TCT when compared to ArODMT is that potentially, only 0.33 equiv of TCT is required to activate 1 equiv of phenol, provided that all three aryl groups on the 2,4,6-triaryloxy-1,3,5-triazine intermediate can be transferred to the Ni catalyst in the oxidative addition step. The reactivity of TCT-activated phenols for C-C couplings has been realized by the Iranpoor group both by first isolating the triaryloxytriazine intermediate or through the in situ activation of the phenol in the presence of sub-stoichiometric amounts of TCT (see Section 6). Notably, this group developed a novel Heck-type coupling method from triaryloxytriazine electrophiles with alkenes (Figure 25) [81].

**Figure 26:**
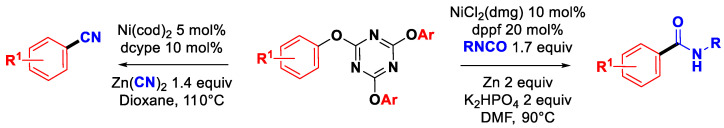
Other C-C couplings developed by the Iranpoor group from triaryloxytriazine.

Although the cross-coupling of a variety of styrene operates in good yields under the optimized conditions, the coupling of acrylates is much less efficient (yields < 30%) regardless of the substation pattern on the phenolic ring. The improved reactivity of triaryloxytriazine when compared to other phenols derived pyridyl ethers was highlighted using *p-*cresol derivatives with styrene under the optimized conditions. TCT derivatives resulted in similar yields (triaryloxytriazine: 77%; diaryloxymethoxytriazine: 77%; and monoaryloxydimethoxytriazine: 67%), and pyridyl and pyrazyl ethers resulted in very low conversion to the desired *p-*methyl stilbene (<15%). Triaryloxytriazine intermediates have also been used by the Iranpoor group in other Ni-catalyzed C-C coupling reactions such as the cyanation [82] or the reductive amidation [83] of triaryl triazine ethers (Figure 26).

## 6. In Situ Activation of Phenols

**Figure 27:**
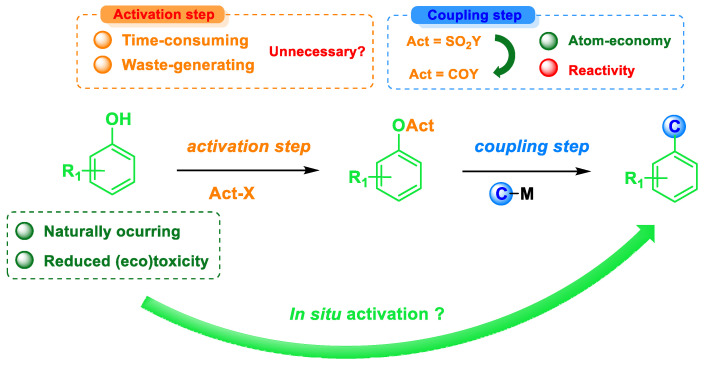
The general concept and features of the in situ activation strategy.

Although small, weakly activated phenol derivatives offer an interesting alternative to aryl sulfonates, especially in improving the atom economy of such C-C coupling reactions, they feature the same drawback as sulfonates in terms of both step-economy and cumulative E factor. Therefore, the design of a cross-coupling method directly from phenols has emerged as a powerful tool to overcome these issues (Figure 27) [84].

**Figure 28:**
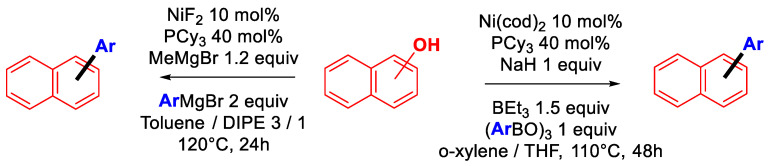
Seminal work on the Kumada and Suzuki–Miyaura of α- and β-naphthols.

Such an approach can be implemented by two different strategies: (a) the one-pot activation/cross-coupling of phenols, which allows for waste reduction by removing the purification step of the preactivated phenol derivative (thus resulting in improved E factor), or (b) the domino activation/cross-coupling, which, although being even more appealing from a green chemistry prospective, remains to date utterly underdeveloped. Initial reports by the Shi group on the direct coupling of naphthols with organomagnesium [85] or organoboronic acids [86] paved the way for improving the sustainability of cross-couplings of phenolic compounds via in situ activation (Figure 28).

However, although being extremely attractive from a green chemistry standpoint, these methods suffer from their limited scope, as only β- (and to a lesser extend α-) naphthols were found to be competent electrophilic partners. Therefore, the development of more versatile C-O activating reagents has attracted many investigations in the context of in situ functionalization of phenols.

### 6.1. Phosphonium Activation (PyBroP)

**Figure 29:**
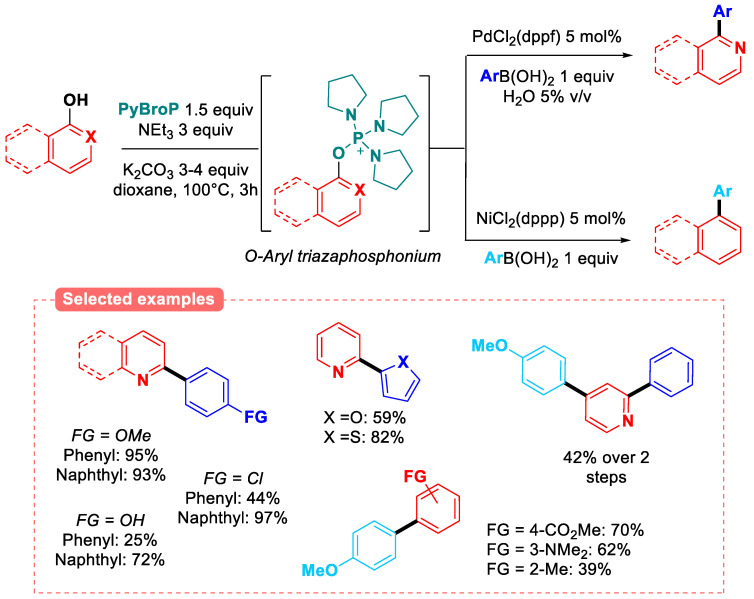
One-pot phosphonium activation/SMC of phenolic compounds.

The use of PyBroP to perform the in situ activation of phenolic compounds has been realized by Han research group via a one-pot phosphonium activation/Suzuki–Miyaura coupling of a variety of pyridinol/quinolinol [87] and phenol derivatives [88] as pro-electrophiles (Figure 29).

In the case of pyridine- or quinoline-based phenols, a palladium catalyst (PdCl_2_(dppf)) was sufficient to perform the oxidative addition in the 2-pyridyl phosphonium intermediate [87], while a nickel catalyst (NiCl_2_(dppp)) was necessary to perform the transformation for both 4-pyridyl and phenyl rings, which do not contain any nitrogen atom [88]. The authors were even able to take advantage of the large reactivity difference of 2- and 4-pyridyl positions to perform the full one-pot phosphonium activation/SMC/SMC to generate diversely substituted 2,4-diarylpyridine with good overall yield (42%).

### 6.2. Trichlorotriazine Activation (TCT)

**Figure 30:**
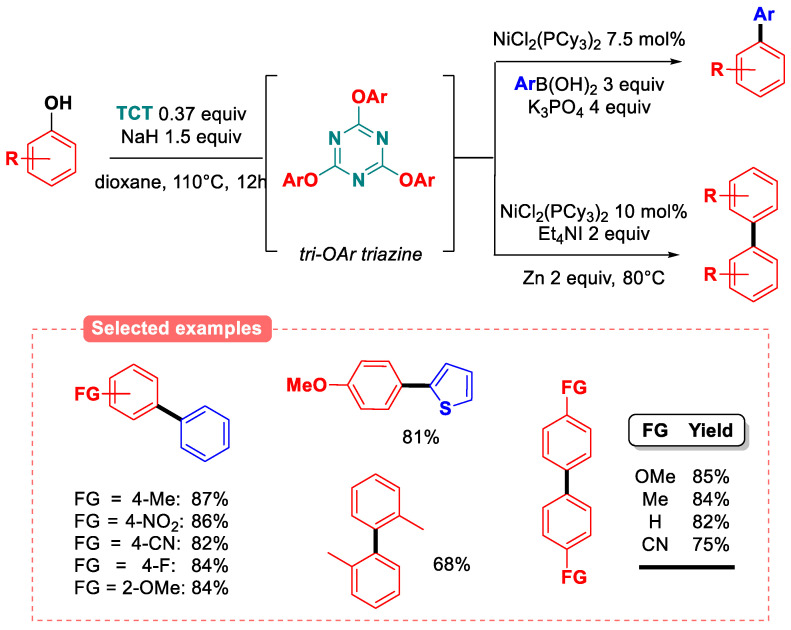
Aryl–aryl couplings of phenols via in situ TCT activation [89,90].

The use of TCT for the in situ phenol activation of phenols has been implemented by Iranpoor and coworkers. In particular, the nickel-catalyzed synthesis of biaryl compounds (Figure 30) was enabled by using a very slight excess of chloropyridine moiety (0.37 equiv of TCT activator), either via SMC [89] or by a reductive homocoupling strategy [90]. Catalyst screening on the SMC reaction of *p-*cresol with phenylboronic acid at 110 °C revealed that a wide range of (diphosphine)nickel (II) dichloride complexes (with phosphines ligands such as dppp, dppf, and even PPh_3_) are efficient pre-catalysts for the transformation, although, once again, the use of NiCl_2_(PCy_3_)_2_ was found to be optimal. However, using ligandless NiCl_2_ as the pre-catalyst did not yield the desired coupling product. The reaction can also be conducted in a variety of ether and aromatic solvents, with 1,4-dioxane being the best. The reaction features a wide functional group tolerance, and ortho-substituted phenols were successfully reacted in these conditions. Furthermore, lignin-based phenols (guaiacol: 84%, vanillin: 82%) as well as steroid derivatives (estrone: 78%) were successfully reacted with phenylboronic acid. Likewise, the reductive homocoupling of phenols operated in similar conditions, with the same nickel catalyst using two equivalents of the zinc powder/tetra(ethyl)ammonium iodide system as the reducing agent at 80 °C.

### 6.3. Dichloroimidazolinedione Activation (DCID)

**Figure 31:**
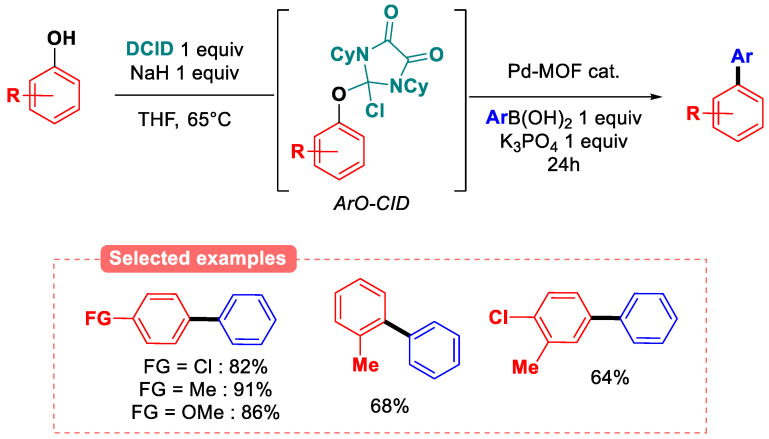
Pd-MOF-catalyzed Suzuki–Miyaura coupling of phenols by in situ DCID activation.

The in situ activation of phenols with dichloroimidazolinedione (DCID) has been realized by means of a heterogeneous palladium/MOF catalysis (Figure 31) under Suzuki–Miyaura coupling conditions [91]. Interestingly, a palladium catalyst was sufficient to perform the oxidative addition in the (aryloxy)chloroimidazolinedione (ArOCID) intermediate. The strong electron-withdrawing effect of the chlorine atom or partial uronium chloride character of the ArO-CID intermediate are noted.

The reaction tolerates phenols diversely substituted in *ortho-*, *meta-,* or *para-*position, and good to excellent yields are obtained in most cases. The heterogeneous catalyst could even be recovered and re-used over four runs without a loss in catalytic activity.

### 6.4. Sulfonate Activation

**Figure 32:**
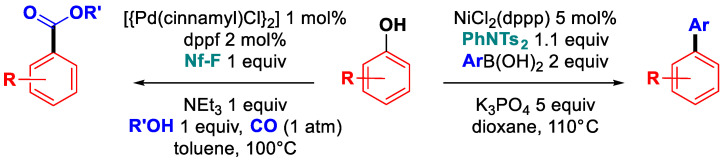
Domino sulfonate activation/C-C coupling: early works.

The design of the C-C coupling of phenols via in situ sulfonate activation has acknowledged many investigations in the last decade. Initial work on the nickel-catalyzed Suzuki–Miyaura coupling of phenols using tosylamines as the activating agent [92] showed that bis(tosyl) aryl and alkyl amines are the most effective ones (Figure 32). N,N-bis(tosyl)aniline gave the best results, affording biaryl compounds in yields ranging from 30% to 93%. The carbonylative coupling of phenols and aliphatic alcohols to generate alkyl benzoates by means of palladium catalysis was also enabled by transient phenol nonaflates generated in situ from the corresponding phenols and nonaflyl fluoride under basic conditions (Figure 32) [93].

**Figure 33:**
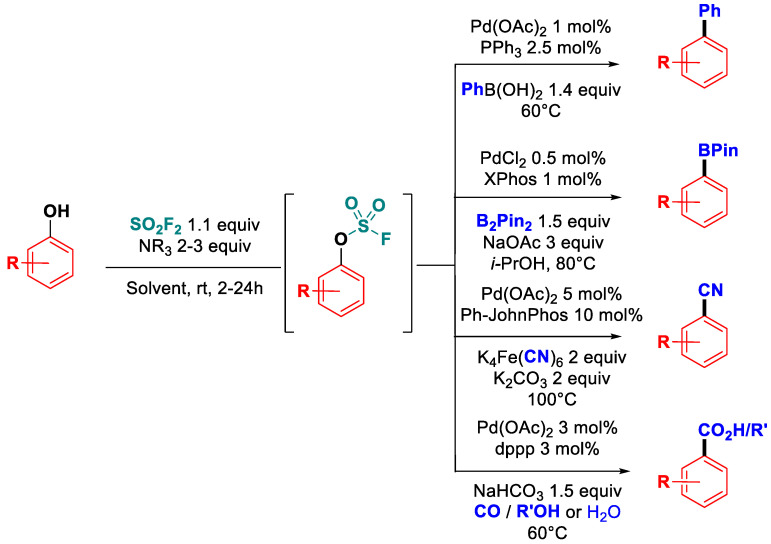
Sulfuryl fluoride-mediated activation of phenols.

Gas sulfuryl fluoride has also been extensively used in this context. Indeed, sulfuryl fluoride readily reacts at room temperature with phenols in a wide range of solvents (such as dioxane, dichloromethane, DMF, or DMSO) using secondary (di-*iso-*propylamine) or tertiary amines (triethylamine) as bases (Figure 33). This approach has proven to be very versatile and was applied to a variety of palladium-catalyzed cross-coupling reactions, including Suzuki–Miyaura coupling [94], Miyaura borylation [95], cyanation [96], and oxy carbonylative couplings to afford benzoic acids and benzoate esters [97]. Remarkably, the palladium-catalyzed Miyaura borylation of phenols with SO_2_F_2_ was found to be extremely functional group tolerant, affording a wide range of aryl pinacolboronic esters bearing both electron-withdrawing or electron-donating substituents in the *ortho-*, *meta-*, and/or *para-*position. In addition, the borylation of natural phenols such as estrone (89% on a gram scale) or vitamin E (22% over two steps) was enabled using this method [95].

### 6.5. Uronium Activation (TFFH)

**Figure 34:**
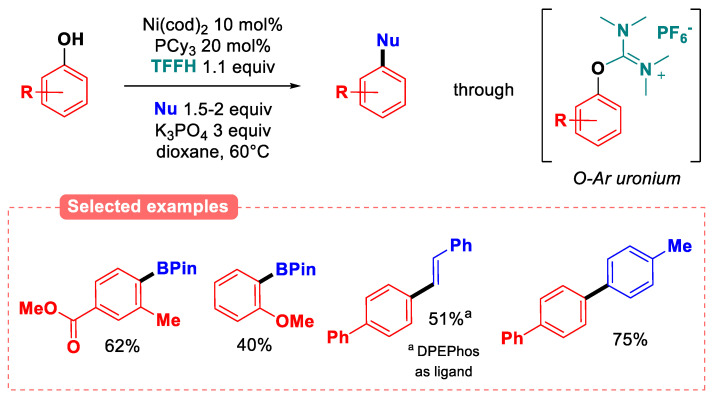
Uronium-activated phenols for various Ni-catalyzed coupling reactions.

More recently, tetramethylformamidinium hexafluorophosphate (TFFH) has been reported as a potent deoxygenating agent for the domino phenol activation/nickel-catalyzed cross-coupling sequence (Figure 34) [98]. The process goes through the formation of an O-aryl uronium species generated from the reaction of the phenol and TFFH with potassium phosphate as base. As potassium fluoride is produced along this process, an arylnickel(II) fluoride species is supposed to undergo transmetalation with the boron reagent. The reaction was found to operate smoothly regardless of the substitution pattern on the phenolic ring.

Furthermore, naturally occurring phenols, such as guaiacol (40%), vitamin E (69%), and pterostilbene (41%), afforded the desired boronic esters in moderate to good yields. The reaction was even extended to Suzuki–Miyaura and Heck coupling reactions under the same conditions.

## 7. Conclusions

In recent years, phenolic derivatives have emerged as a replacement for organic halides in cross-coupling reactions due to their natural availability, low toxicity, and low cost. They are economically attractive and represent a more environmentally friendly alternative to existing electrophiles. However, phenols have a low reactivity towards oxidative addition, requiring prior activation of the hydroxyl group to form more reactive intermediates. To activate the hydroxyl group, various methods involving O-aryl ester, O-aryl carbonate, O-aryl carbamate, and O-aryl ether derivatives have been developed. These compounds have proven to be viable electrophiles for the design of greener cross-coupling reactions, especially via the use of nickel-based catalysts. Concerning the coupling of O-carbonyl (esters, carbonates, and carbamates) and O-pyridyl phenol derivatives, Ni^0^(PCy_3_)_2_ was found to be a very versatile catalyst, affording the coupling product in high yields at moderate temperatures (ca 100 °C for most methods). However, in the case of less-activated anisole C-O bonds, a bulky NHC ligand (ICy) was often required to perform the oxidative addition step. In the last decade, the in situ activation of phenols using various deoxygenating reagents has emerged as a very promising strategy to further improve the sustainability of the coupling process. However, such a strategy is yet unfortunately limited to toxic and/or poor-atom-economic and waste-generating stoichiometric C-O bond activators. In addition, the coupling of naturally occurring phenol backbone is mostly limited to estrone derivatives [71,72,73,74,95], as very few lignin-based phenols have been employed in these coupling processes [89,98]. Finally, the design of more reactive nickel catalyst might allow us to increase further the greenness of such coupling processes by lowering the catalyst loading and/or the reaction temperature. Thus, despite the spectacular improvement that has been made in this area in the last two decades, the design of greener and safer C-C coupling protocols using phenols still poses significant challenges and will surely be a thoroughly investigated field in the upcoming years.
